# The effects of arthritis gloves on people with Rheumatoid Arthritis or Inflammatory Arthritis with hand pain: a study protocol for a multi-centre randomised controlled trial (the A-GLOVES trial)

**DOI:** 10.1186/s12891-017-1583-4

**Published:** 2017-05-30

**Authors:** Yeliz Prior, Chris Sutton, Sarah Cotterill, Jo Adams, Elizabeth Camacho, Nazina Arafin, Jill Firth, Terence O’Neill, Yvonne Hough, Wendy Jones, Alison Hammond

**Affiliations:** 1 Centre for Health Sciences Research (OT), University of Salford, Frederick Road, L701 Allerton Building, Salford, Greater Manchester M6 6PU UK; 2 Mid Cheshire NHS Trust, Leighton Hospital, Leighton Crewe, UK; 3 Lancashire Clinical Trials Unit, UCLAN, Preston, UK; 4 Centre for Biostatistics, School of Health Sciences, University of Manchester, Manchester, UK; 5 Health Sciences, University of Southampton, Southampton, UK; 6 Centre for Health Economics, Division of Population Health, Health Services Research, and Primary Care, School of Health Sciences, University of Manchester, Manchester, UK; 7 Pennine Musculoskeletal Partnership Ltd, Oldham, UK; 8 Arthritis Research UK Centre for Epidemiology, University of Manchester & NIHR Manchester Musculoskeletal Biomedical Research Unit, Central Manchester University Hospitals NHS Foundation Trust, Manchester Academic Health Sciences Centre, Manchester, UK; 9 St Helens and Knowsley Teaching Hospitals NHS Trust, St Helens Hospital, St Helens, UK; 10 Patient Research Partner, Manchester, UK

**Keywords:** Arthritis gloves, Rheumatoid arthritis, Inflammatory arthritis, Occupational therapy, Hand pain, Hand stiffness, Hand swelling, Compression gloves, Splints

## Abstract

**Background:**

Arthritis gloves are regularly provided as part of the management of people with rheumatoid arthritis (RA) and undifferentiated (early) inflammatory arthritis (IA). Usually made of nylon and elastane (i.e. Lycra**®**), these arthritis gloves apply pressure with the aims of relieving hand pain, stiffness and improving hand function. However, a systematic review identified little evidence supporting their use. We therefore designed a trial to compare the effectiveness of the commonest type of arthritis glove provided in the United Kingdom (Isotoner gloves) (intervention) with placebo (control) gloves (i.e. larger arthritis gloves providing similar warmth to the intervention gloves but minimal pressure only) in people with these conditions.

**Methods:**

Participants aged 18 years and over with RA or IA and persistent hand pain will be recruited from National Health Service Trusts in the United Kingdom. Following consent, participants will complete a questionnaire booklet, then be randomly allocated to receive intervention or placebo arthritis gloves. Within three weeks, they will be fitted with the allocated gloves by clinical specialist rheumatology occupational therapists. Twelve weeks (i.e. the primary endpoint) after completing the baseline questionnaire, participants will complete a second questionnaire, including the same measures plus additional questions to explore adherence, benefits and problems with glove-wear. A sub-sample of participants from each group will be interviewed at the end of their participation to explore their views of the gloves received. The clinical effectiveness and cost-effectiveness of the intervention, compared to placebo gloves, will be evaluated over 12 weeks. The primary outcome measure is hand pain during activity. Qualitative interviews will be thematically analysed.

**Discussion:**

This study will evaluate the commonest type of arthritis glove (Isotoner) provided in the NHS (i.e. the intervention) compared to a placebo glove. The results will help occupational therapists, occupational therapy services and people with arthritis make informed choices as to the value of arthritis gloves. If effective, arthritis gloves should become more widely available in the NHS to help people with RA and IA manage hand symptoms and improve performance of daily activities, work and leisure. If not, services can determine whether to cease supplying these to reduce service costs.

**Trial registration:**

ISRCTN Registry: ISRCTN25892131 Registered 05/09/2016

**Electronic supplementary material:**

The online version of this article (doi:10.1186/s12891-017-1583-4) contains supplementary material, which is available to authorized users.

## Background

Rheumatoid arthritis (RA) is a chronic inflammatory condition causing joint pain, swelling (synovitis), stiffness and muscle loss around affected joints [1]. It affects 1% of the population, and twice as many women as men. Peak onset is in the 40-60 age group, it is incurable and causes significant disability if untreated [2, 3]. RA is managed with disease modifying anti-rheumatic drugs (DMARDs), rehabilitation and self-management education. People with persistent synovitis, where other pathologies are ruled out (i.e. undifferentiated inflammatory arthritis (IA), who do not yet meet the criteria for RA [4]), also require specialist care and DMARDs, and are thus treated as if they have RA [1]. Most people with IA and RA have symptoms in both hands, resulting in problems with everyday activities, work and leisure. Many experience frustration and distress because of hand pain, stiffness and disability. A third are work disabled within 5 years [1].

Arthritis gloves are commonly provided by Rheumatology occupational therapists to people with IA or RA. These are worn for pain relief during the day or night and to improve hand function during the day. We surveyed Rheumatology occupational therapists,﻿﻿identifying that most provide arthritis gloves [5]. Provision varies considerably between occupational therapists but averages a third of the patients they see. The most common make of glove provided is the mid-finger Isotoner glove (i.e. finger tips exposed to aid hand function) and the next commonest are Jobskin and Norco oedema gloves [5, 6]. Provision of gloves appears to have risen following a randomised controlled trial (RCT) demonstrating an alternative treatment (resting splints) was ineffective in early RA [7]. (Glove photographs are in Additional file 1: study information).

Arthritis gloves are usually made of nylon and elastane (i.e. Lycra®). They are thought to impact on hand symptoms through providing compression (pressure) and/or warmth. Some models apply both and others warmth only. Isotoner gloves, containing 20% elastane, exert the highest pressure of those makes available, at 23-32 mmHg [8]. Other makes exert less as these contain less elastane (e.g. the Jobskin classic oedema gloves exert 15-25 mm Hg and contain 11% elastane [9]). When properly fitted (to be a “snug fit”), the gloves apply controlled pressure to the hand [10]. The pressure is thought to: (a) remove extracellular fluid, thus reducing pain, stiffness and improving finger motion; and (b) increase blood flow and consequently warmth, reducing pain [10, 11]. The glove material also provides warmth, contributing to pain relief. Makes of arthritis gloves, which specifically apply pressure, are termed compression or oedema gloves. It is unclear what a therapeutic level of pressure is, as this has not been identified through physiological studies. However, based on pressure information from manufacturers, therapists consider this is between 15-32 mmHg. We also hypothesize that arthritis gloves provide tactile feedback to the glove wearer reminding them to take more care of their hand joints. Potentially, all models of glove (however much pressure and/or warmth they apply) may be acting through this mechanism. This effect was hypothesized in a pilot clinical effectiveness and efficacy trial of thumb splints [12].

Despite their widespread use, evidence for the effectiveness of arthritis gloves is inconclusive. In a recent systematic review, we identified only four trials evaluating arthritis gloves. Trials were small and results inconclusive [13]. We therefore developed this randomized controlled trial to evaluate the effectiveness and cost-effectiveness of the most commonly used arthritis glove (i.e. Isotoner) in people with RA or IA.

### Feasibility study

We conducted a feasibility study among patients with IA or RA (*n* = 39) in 10 Occupational Therapy departments in Northwest England evaluating mid-finger Isotoner gloves (as these are the commonest type provided and apply the highest pressure). During this study we standardised arthritis glove eligibility criteria, glove treatment protocols (including the arthritis glove patient information sheet) with participating therapists (North-West College of Occupational Therapy Specialist Section in Rheumatology: NWCOTSS-R arthritis glove protocol [14]. The results of the study are reported elsewhere [15].

#### Objectives

The primary objective is to assess whether there is a clinically important difference in self-reported dominant hand pain during daytime activity between participants with RA or IA receiving intervention gloves (Isotoner gloves) in addition to usual care compared to participants receiving placebo gloves (Jobskin classic oedema gloves fitted at least one size too big to ensure similar warmth is provided but minimal pressure only is applied), plus usual care.

The secondary objectives are to:

i. assess the effectiveness of intervention gloves, relative to placebo gloves on self-reported: non-dominant hand pain during activity; dominant and non-dominant nocturnal hand pain; hand pain during the day at rest; hand stiffness; hand joint swelling; and hand function.
ii. evaluate the cost-effectiveness of arthritis gloves compared with placebo gloves, taking into account the cost of the gloves and other healthcare resources used by participants.
iii. explore participants’ views of: the effects of arthritis (intervention) and placebo gloves on hand symptoms, function, and their daily lives; acceptability of glove wear; and how and when they prefer to use these.

#### Trial design

The A-GLOVES trial is a pragmatic, patient-blinded, multi-centre, superiority randomised parallel group trial of intervention gloves compared to placebo gloves in people with RA or IA and persistent hand pain affecting their ability to do daily activities. Analysis will be on an intention-to-treat basis. Ethical approval for this study has been obtained from the North of Scotland Local Research Ethics Committee [15-NS-0077]. The study protocol was developed using the SPIRIT guidelines [16].

## Methods

### Study setting

Study participants will be recruited from Rheumatology, Occupational Therapy and Hand Therapy departments in 23 hospitals across 17 NHS Trusts in England and Scotland in the United Kingdom (UK), as arthritis gloves are most commonly provided by rheumatology occupational therapists in secondary care.

### Eligibility criteria

#### Inclusion criteria

Patients eligible for the trial must comply with all of the following at randomization:

1. Aged ≥18 years
2. Diagnosed with RA or IA by a Rheumatology Consultant
3. Have persistent pain in the proximal interphalangeal (PIPs) and/or metacarpophalangeal (MCP) joints causing either
  i) difficulty using their hands during the day (for day wear of gloves) or
  ii) disturbed sleep (for night wear of gloves) or
  iii) limited ability to use their hands when waking/in the morning (for night wear of gloves)
  4. Willing to wear arthritis gloves and participate in the trial
  5. Able to read and understand English and,
  6. Can provide informed consent.

#### Exclusion criteria

People will be excluded from the study who have:

1. Been diagnosed with other rheumatic conditions, such as gout, psoriatic arthritis, ankylosing spondylitis, connective tissue disorders (systemic lupus, systemic sclerosis), resulting in inflammatory arthritis in the hand/s
2. Severe Raynaud’s disease or other circulatory disturbances in the hand
3. Severe neuropathies (nerve damage) in the hand
4. Severe hand deformities
5. Any contraindications to wearing the gloves (e.g. eczema, infections, broken skin)
6. Previously worn arthritis gloves.

### Interventions

#### Occupational therapist training in glove provision

Interventions will be delivered by 27 National Health Service (NHS) Rheumatology occupational therapists (≥Band 6 i.e. clinical specialists). Participating therapists must attend a one-day clinical trial training programme delivered by expert Rheumatology occupational therapists and the research team. This will include: trial background; key study procedures; and practice in providing the intervention and placebo gloves in a standardised manner. The A-GLOVES Occupational Therapy Glove Provision Manual, developed by the research team with the NW-COTSS-R, will be followed when fitting these gloves [17].

In addition to the training day, the Trial manager will conduct site visits to ensure all Principal Investigators, research facilitator/s (i.e. nurses/other staff employed in the NHS to assist with recruitment into trials) and occupational therapists involved in the study understand how to explain the study and arthritis gloves appropriately, to ensure participants are not unblinded to the intervention.

#### Glove fitting

The intervention group will receive correctly fitted mid-finger length Isotoner arthritis gloves. The placebo group will receive mid-finger length Jobskin classic oedema gloves fitted at least one size too large, to ensure they do not apply therapeutic levels of compression. When fitting gloves, the occupational therapist will measure participants’ MCP circumference to determine the glove size required. Therapists will also use their clinical judgement to determine appropriate fit. Usually, patients requiring gloves receive these for both hands as their hand pain and/or swelling is bilateral. However, if patients have unilateral pain and/or swelling, they are provided with a glove only for the affected hand.

#### Additional Interventions

All participants (in both intervention and placebo groups) will receive a booklet about hand self-management: “Looking After Your Joints when you have arthritis” [18]. This booklet is widely provided in clinical practice. They will also receive an information sheet about hand exercises, based on the Strengthening And Stretching For Rheumatoid Arthritis of the Hand (SARAH) trial hand exercise programme for RA [19, 20]. During the 12 weeks, participants will only receive brief training in joint protection and hand exercises, and this will not use cognitive-behavioural approaches. Most departments do not normally offer behaviourally based joint protection and/or hand exercise programmes (or where these exist, such programmes usually have waiting lists), thus participants are not being disadvantaged.

#### Modifications

In some instances, a glove may not be fitted if enlarged PIP joints or finger deformity prevent this. For those participants with an MCP circumference greater than 23.5 cm (or fingers/hands too large in other respects), no gloves will be fitted, as an appropriately large size is not available from manufacturers. If the participant cannot be fitted with gloves, they still remain in the trial, in line with “intention-to-treat.”

#### Adherence

Recommendations for when to wear gloves will be based on individual needs; most, but not all, patients will be provided with gloves for both hands. Most people experience hand pain during the day and are recommended to wear gloves during activity. Those experiencing hand pain at night, which interferes with sleep, will also be recommended to wear gloves at night. Gloves are not recommended to be worn continually. All participants will receive written information about glove wear and care, using the sample information sheet in the A-Gloves Occupational Therapy Glove Provision Manual [17].

To check for correct glove fit and any problems, occupational therapists will either: within two to four weeks of glove provision offer a review appointment (in person or by telephone); or ask the participant to get in contact if experiencing problems, if this is their normal departmental policy. At the review appointment, participants will be reminded about their glove wear regimen and the need to continue to wear the gloves until the 12-week follow-up questionnaire is completed. At the end of their trial participation, they will be contacted with further instructions about future glove wear.

Adherence to glove wear will be assessed in the 12-week follow-up questionnaires, by asking participants to describe their glove wear for right and left hand gloves. This will include, over the last four weeks, the: average time worn during the day and/or at night; and the average number of days per week gloves were worn. The participant will also be asked to state whether they have obtained arthritis gloves from elsewhere (if they did so, as it is possible for patients to purchase gloves in shops and on-line) and glove wear related to these (if applicable).

#### Concomitant care

Occupational therapists are asked not to provide resting, wrist, finger or thumb splints or any other occupational therapy interventions (apart from joint protection and hand exercises) to participants whilst they are in the trial (i.e. during their 12 weeks participation). However, participants are permitted to attend Physiotherapy for lower limb interventions if required. Data on participants’ use of concomitant care is collected via the 12-week questionnaire.

### Outcomes

The primary outcome measure is ‘hand pain during moderate activity’ which was considered the most important outcome by glove-users in our feasibility study. This is measured as hand pain in the dominant hand during the day, on a typical day, when doing moderate hand activities, e.g. housework, cooking, Do-It-Yourself, gardening. In our feasibility study, 2% of participants received one glove for their non-dominant hand only. It is therefore possible that the primary outcome cannot be collected in a small number of participants in this trial.

Secondary outcome measures are hand pain when resting and at night; stiffness; self-reported hand condition; hand function; disability; and resource use and costs to measure cost-effectiveness of glove provision (Table 1).

**Table 1:** Content of the Baseline and 12-week follow-up questionnaires

Concept	Measurement method	Details	0-wks	12-wks
*Demographic and Condition Information*	Date of birth		✓	
	Gender		✓	
	Time since RA or IA symptom onset		✓	
	Time since RA or IA diagnosis		✓	
	Employment status		✓	
	Marital status		✓	
	Living status (alone; or with family/significant others)		✓	
	Medication regimen (i.e. what drugs do they take for their arthritis);		✓	
	Whether received a steroid injection/oral steroid in the last 6 weeks		✓	
	Hand dominance (i.e. whether they consider this to be right, left or both).		✓	
*Primary outcome*	Hand Pain during activity	0-10 (0 = no pain/10 = severe pain) point numeric rating scale of hand pain in the dominant hand during the day [21]	✓	✓
*Secondary outcomes*	Hand Pain	0-10 (0 = no pain/10 = severe pain) a) during a typical day during activities in the last week in the non-dominant hand; b) when resting- separately for the dominant and non-dominant hands; and c) at night –separately for the dominant and non-dominant hands.	✓	✓
	Stiffness	Measured separately for the dominant and non-dominant hands: a) Patient self-reported duration of early morning stiffness affecting the hands (hours/min) b) 0-10 point numeric rating scale of hand stiffness (no (0) and severe (10) hand stiffness)	✓	✓
	Self-reported hand condition	a five point rating scale of very severe/severe/moderate/good/very good.	✓	✓
Hand Function	The Measure of Activity Performance of the Hand (MAPHAND) [22, 23]	a self-reported measure of 18 items of performing daily activities with the hands	✓	✓
	The Michigan Hand Outcomes Questionnaire (MHQ) [24, 25]	assesses right and left hands separately: physical status of the hand (movement, strength, sensation: 5 items); daily activities performed with the hands/arms (5 right and left; 7 bilateral); impact of their condition on their normal activities (5 items); pain frequency, severity and impact (5 items); perceived appearance of their hands (4 items); satisfaction with hand abilities (6 items)	✓	✓
Disability	The Health Assessment Questionnaire [26]	24 items of daily function	✓	✓
Economic analysis	EQ5D-3 L [27, 28]	5-items Scale (Mobility; Self-care; Usual activities; Pain/Discomfort; Anxiety/Depression	✓	✓
	Your use of NHS and social services	a) Any planned hospital overnight stays in the last 3 months b) List of planned admissions	✓	✓
	Your use of hospital out-patient appointments	a) Any planned hospital outpatient appointments lasting 4 h or less in the last 3 months b) If yes, department, speciality and number of appointments	✓	✓
	Your use of day hospital appointments	a) Any day or hospital outpatient lasting more than 4 h but not overnight during the last 3 months b) If yes, department, speciality and number of appointments	✓	✓
	Your use of accident and emergency services	a) Any A&E attendance in the last 3 months b) If yes, the number of visits did not lead to hospital admission c) Were admitted into a hospital as an in-patient from the A&E d) If yes, department, reason for admission, where and when admitted	✓	✓
	Your use of primary and community based health services	a) Use of services such as GP, Practice nurse, Nurse, Counsellor in the last 3 months b) If yes, number of visits to each	✓	✓
	Your use of primary and community based health services	a) Use of services such as, occupational therapy, Physio, Care worker, Home help, Social worker, Other in the last 3 months b) If yes, number of visits to each	✓	✓
Medication	Current medication for RA/IA			✓
	Any steroid injection/oral steroids started in the last 12 weeks	Yes/No		✓
	If yes, the date of the injection/started taking oral steroids	DD/MM/YY		✓
Health Status	Your own health state today	Measured by a 0-100 vertical scale (0 = worst imaginable state & 100 = best imaginable health state)		✓
Additional outcomes	Any other upper limb occupational therapy or physiotherapy treatment received in the last 12 weeks	Type of treatment received		✓
	Whether purchased or obtained from elsewhere, any other “arthritis” gloves.	Yes/No		✓
	If yes, what type these were			✓
	How their hands are in comparison to 12 weeks ago, i.e. before receiving gloves	(much better/better/no change/worse/much worse)		✓
	Concurrent use of any resting, wrist, finger or thumb splints			✓
	Adherence to glove wear	During the day and at night for right/left hand gloves; average time worn at night/during the day; average number of days per week gloves have been worn		✓
	Whether participants considered gloves provided any benefit	Yes/No		✓
	Whether they will continue to wear the gloves provided	Yes/No		✓
	If they considered the gloves of any benefit, what were these			✓
	Any problems encountered when wearing gloves	Freetext		✓

### Participant timeline

**Fig. 1:**
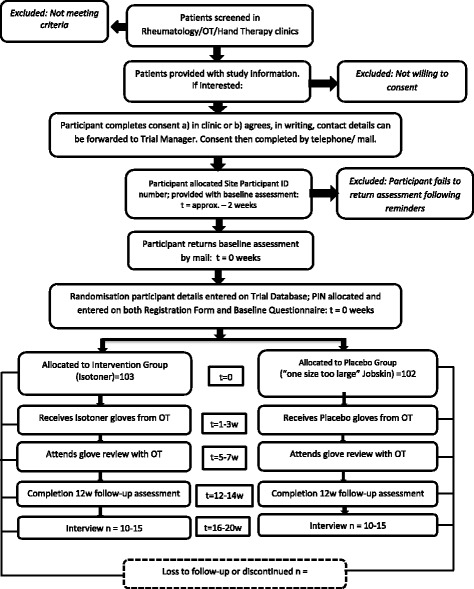
A-GLOVES trial: flow of participants

**Fig. 2:**
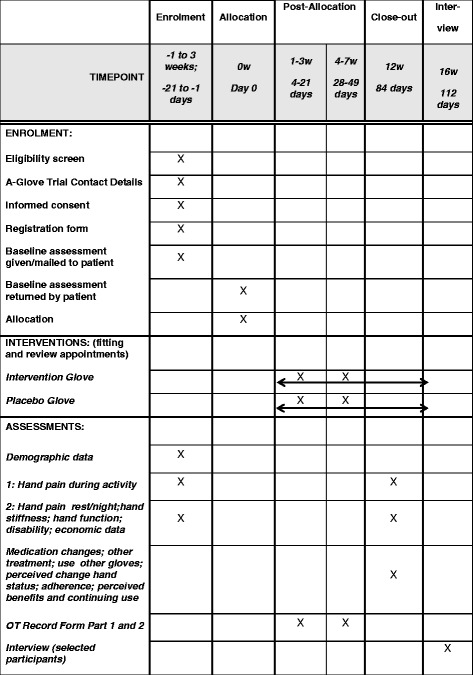
SPIRIT flowchart: schedule of enrolment, interventions and assessments

Participants will complete a baseline questionnaire following consent and prior to the randomisation at week zero. Within three weeks of randomisation, an occupational therapy glove fitting appointment will be arranged to ensure there is sufficient length of time to wear the gloves prior to the 12-week follow-up. Two to four weeks after glove fitting (dependent on each departments’ usual practice), participants will attend an occupational therapy review appointment, either in person or by telephone, as per departmental policy. Participants will receive the follow-up questionnaire at 12-week following the date of baseline questionnaire completion. (See Figs. 1 and 2).

#### Data collection

The baseline questionnaire includes: demographic factors i.e. age, gender, living situation, number of dependents living with them, and employment status; and condition specific factors, i.e. duration of their symptoms, time since diagnosis, current medication regimen and whether they have had a steroid injection/started on oral steroids within the last six weeks.

Both the baseline and the 12-week follow-up questionnaire include:

The primary outcome:

1. Hand pain: measured on a numeric rating scale (NRS) of hand pain in the dominant hand during the day when doing moderate hand activities. The anchor points are no pain (0) to severe pain (10). The pain NRS is a widely-used outcome measure in RA clinical trials. During development and psychometric testing of a patient-reported outcome measure, it was identified that participants had a strong preference for completing NRS over visual analogue scales. This study also identified test-retest reliability of pain (on movement and at rest) were between r_s_ = 0.70 to 0.72. The pain NRSs had significant correlations (*p* < 0.001) with SF36v2 Bodily Pain scales (r_s_ -0.69 to -0.77) [21] (Table 1).
  And secondary outcomes including:
2. Stiffness: measured through a) Patient self-reported duration of early morning stiffness affecting the hands (hours/minutes); b) 0-10 point numeric rating scale of hand stiffness (with anchor points of no (0) and severe (10) hand stiffness) - separately for the dominant and non-dominant hands.
3. Self-reported hand condition: a five-point rating scale of very severe/severe/moderate/good/very good.
4. Hand function: measured by the Measure of Activity Performance in the Hand (Map-HAND), which has been shown to be unidimensional and have good reliability and validity in a British RA population [22, 23] and the Michigan Health Questionnaire (MHQ), which assesses for the right and left hands separately: physical status of the hand (movement, strength sensation: 5 items); daily activities performed with the hands/arms (5 right and left; 7 bilateral); impact of their condition on their normal activities (5 items); pain frequency, severity and impact (5 items); perceived appearance of their hands (4 items); satisfaction with hand abilities (6 items) and also has good reliability and validity [24, 25].
5. Disability: the Health Assessment Questionnaire (24 items of daily function) [26]
6. Health-related quality of life (HRQoL) is measured using the standardised five-item EuroQoL, 3-level version (EQ-5D-3 L) [27, 28], which is recommended by National Institute of Health and Clinical Excellence (NICE) for economic evaluations in clinical trials and has proven responsiveness, reliability, and validity in trials of interventions for RA [29, 30].

The outcome measures within the baseline and 12-week questionnaire are also listed in Table 1.

#### Patient interviews

At 14 weeks, participants (*n* = 10–15 from each group) will be purposively selected to participate in a semi-structured, face-to-face or telephone interview to investigate their views on: benefits or negative effects of glove wear (including when at work for those who are employed); glove appearance, quality, comfort, ease of applying; and willingness to buy gloves in future. Purposive sampling will be based on: 1:3 male to female ratio (as per the distribution of RA in population), and a range of ages, baseline hand pain (mild/moderate/or severe) and 12-week self-reported levels of adherence with glove wear. The semi-structured interview schedule is outlined in Table 2.

**Table 2:** A-GLOVES semi-structured qualitative interview schedule

A-GLOVES Trial: Qualitative Semi-structured Interview Schedule	
Opening/Main Question	
“Having worn the arthritis gloves for up to 12 weeks, could you tell me about any negative or positive effects these have had on your hand pain and hand problems?”	
The following prompts may be used to expand on the answers given:	
1	What effect did they have on your hand pain, hand stiffness and ability to use your hands?
2	Were there any particular activities you found they helped with? [For example: personal care; household activities; leisure/social activities; driving; work].
3	Were there any particular activities you found they did not help with? [For example: personal care; household activities; leisure/social activities; driving; work].
4	How did you find wearing them?
5	How was it to put them on and off your hands?
6	Was there anything about the gloves or their effects which you think helped/hindered your hand pain and hand problems?
7	If they were helpful: when did you find them helpful to wear: either in the day or at night (or both)?
8	If they were not helpful: when did you find them unhelpful to wear: either in the day or at night (or both)?
9	For those employed: Have you used them at work? If yes, were they helpful? And in what ways? If not helpful, why was this?
10	Did you have any problems wearing the gloves?
11	What did you think of the gloves appearance?
12	What did you think of the quality of the gloves you were given?
13	How did you find cleaning them?
14	Would you consider buying them in the future?
15	Would you change anything about them to make it better for your use? (e.g. colour, texture, amount of pressure applied, size, length)

All interviews will be audio recorded, transcribed verbatim and thematically analysed by three researchers to increase the validity of findings.

### Sample size

This was calculated using data from the feasibility study. Minimal clinically important differences for pain scales in RA are estimated as 1.1 points on a 0–10 scale [31, 32]. The mean change in hand pain score during activity (measured four weeks post-intervention) was -1.03 (SD 2.22). As the SD from the feasibility data might be an underestimate, the 80% upper one-sided confidence limit of the estimated SD, i.e. 2.48 was used. To identify a 1.1 point difference, SD = 2.48, p = 0.05 and 80% power, 80 participants are required per group. Allowing for a 20% attrition (i.e. non-return of 12 week questionnaire and a small number (up to 2%) not receiving a glove for their dominant hand (because they only require a glove for their non-dominant hand, and therefore not providing primary outcome data), we intend to recruit 205 participants.

### Recruitment

At each participating site a Principal Investigator (PI) (senior occupational therapist/consultant rheumatologist) will be identified, to be responsible for identification, recruitment, consent and provision of baseline questionnaires, along with adherence to the study and treatment protocols, following Good Clinical Practice Guidelines.

**Fig. 3:**
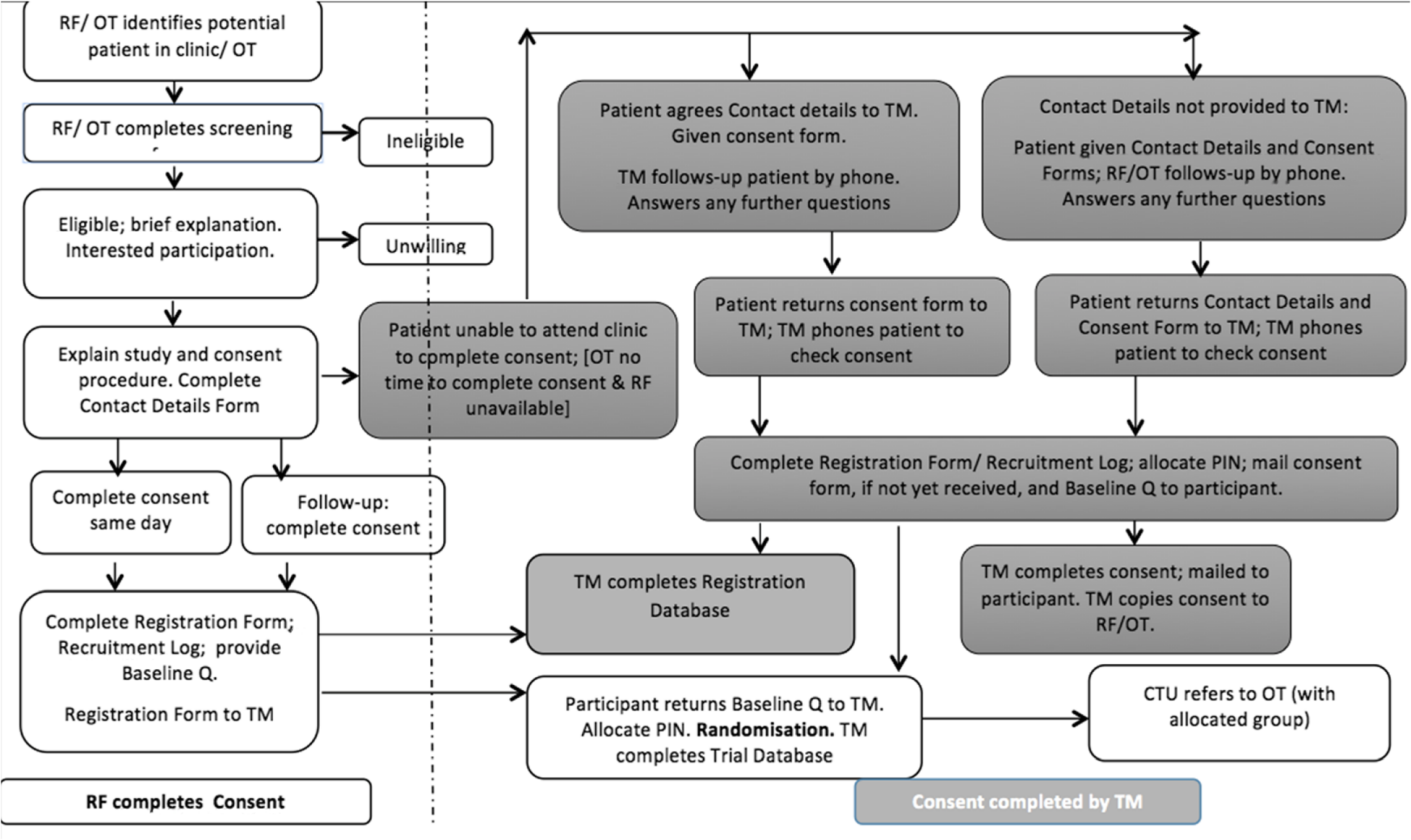
Recruitment and consent flowchart: rheumatology clinic/occupational therapy

Members of the health care team and occupational therapists at participating sites will identify adult patients with RA or IA and persistent hand pain during the patients’ Rheumatology, occupational therapy or hand therapy appointment. Either a research facilitator or occupational therapist will then screen patients for eligibility using the A-Gloves Trial Eligibility Screening Form. (See Fig. 3 for recruitment procedure). All eligible patients will be provided with a study explanation and information pack. (See Additional file 1).

**Fig. 4:**
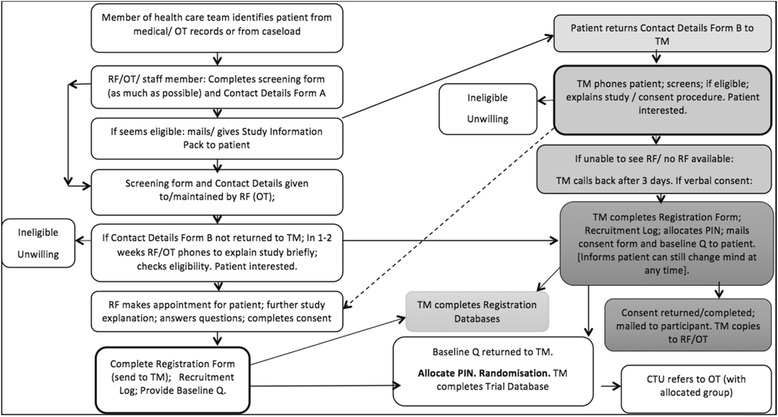
Recruitment flowchart via medical/OT records/health care staff and study information pack mailed/provided to patient

If a site is encountering difficulty screening sufficient numbers in clinics, then potential participants will be identified from medical or occupational therapy records by members of the health care team. The patient will then be mailed a Study Information Pack by the research facilitator or occupational therapist. (See Fig. 4 for recruitment procedure).

At the screening stage, participants identified as having had a steroid injection or started oral steroids in the last six weeks, but who otherwise meet the inclusion criteria, will not be consented but will be re-screened six weeks after the date of steroid injection/starting oral steroids to re-check trial eligibility. Steroids can reduce hand pain and thus would be a confounding factor in evaluating glove effectiveness. If at six weeks they are still eligible, they will be re-approached for consent.

Participants may receive a steroid injection or start oral steroids following consent. If this occurs between consent and glove fitting, the occupational therapist will identify this at the glove fitting appointment and defer glove fitting for six weeks from the date of injection/steroid start. It is not anticipated that this will be a common event. If the participant starts steroids following glove provision and their hands are still symptomatic, they will be advised to continue glove wear as prescribed, as this is a pragmatic trial. If the patient reports that their hands are no longer causing them pain or discomfort they will be advised not to wear the gloves. However, they will remain in the trial and complete the 12-week questionnaire, as we will be conducting an intention to treat analysis.

Following consent, by the research facilitator or occupational therapist, participants will be provided with a baseline (i.e. 0 weeks) questionnaire to complete and return in a Freepost envelope to the trial manager. On receipt of the completed questionnaire, the trial manager will enter their details into a web-based MACRO database (managed by Lancashire Clinical Trials Unit [CTU]) and request that the unblinded CTU staff perform the assignment of the intervention.

### Assignment of intervention

#### Allocation and sequence generation

Participants will be randomly assigned to either the intervention or placebo glove group with a 1:1 allocation generated and delivered by ‘Sealed Envelope,’ an online, central randomisation service (www.sealedenvelope.com). The randomisation schedule will be stratified by whether or not the participant has had a change in or received new medication (specifically DMARDs or biologics) within the last three months, using permuted blocks of random sizes.

#### Concealment mechanism

The block sizes or schedule will not be disclosed to the trial manager, occupational therapists or research facilitators to ensure concealment. Randomisation will occur only once the trial manager confirms a participant’s eligibility, consent and that they have completed the baseline questionnaire.

#### Implementation

Within three working days of notification by the trial manager, unblinded CTU staff will perform the randomisation and securely e-mail a referral for either intervention or placebo gloves to the relevant site. Within three weeks of referral, the occupational therapist will make an appointment and provide the participant with the appropriate gloves.

### Blinding

Due to the nature of the intervention it will not be possible for therapists to be blinded to group allocation. As most sites will only have one Rheumatology occupational therapist, both intervention and placebo gloves will commonly be provided by the same occupational therapist. During the glove training and site monitoring visits, the importance will be emphasised of not divulging to the participant whether they are receiving an intervention (arthritis glove) or a placebo glove. Therapists will be asked not to use the term “compression” glove (an alternative name for arthritis gloves in clinical practice) in any participant interaction, as this may unblind patients as to whether they are receiving an intervention or placebo glove. Participants will be kept blinded to group allocation by describing the study throughout as a comparison of two types of arthritis glove and not divulging the differences between these.

The trial manager will remain blinded to group allocation until the participant has completed and returned their 12-week questionnaire and the data been verified at the CTU. The trial manager will then be unblinded to group allocation for some participants in order to complete interviews with participants (*n* = 24–30).

Data co-ordination and data entry staff at the CTU, responsible for baseline and 12 weeks questionnaires management, will be blinded to group allocation. However, data entry for the Treatment Records (which identify the group allocation) will be conducted separately by the CTU Trial Management Team only, to avoid other CTU staff becoming unblinded. Statisticians and the health economist will be blinded to group allocation until analysis is complete.

#### Emergency unblinding

Not applicable as serious adverse events are not known to occur in clinical practice.

### Data collection methods

Outcomes will be collected via self-reported questionnaires at baseline (i.e. prior to randomisation) and 12 weeks later. Glove provision will usually occur within three weeks of randomisation and referral to occupational therapy. Thus, at follow-up, most participants will have worn gloves for about nine weeks. Feedback from glove users indicates that they normally experience any benefits within a short-time of commencing glove-wear.

The trial manager (baseline) and CTU (12 weeks) will monitor return of all questionnaires and the quality of data.

- **At 1 week** after questionnaire provision/mailing, if the questionnaire is not yet returned, the trial manager [baseline questionnaire] or CTU [12-week questionnaire] will telephone/text/e-mail (as applicable) to remind the participant to return their questionnaire.
- **At 2 weeks** after questionnaire provision/mailing, if the questionnaire is not yet returned, the trial manager [baseline questionnaire] or CTU [12-week questionnaire] will mail a reminder letter and a further copy of the relevant questionnaire (with Freepost envelope).
- **If the 12 week questionnaire is not returned by week 16,** the CTU will inform the trial manager who will then telephone the participant to obtain a minimal data set (i.e. at least dominant hand pain during moderate activity and if gloves are being worn (yes/no)). If possible, as much of the following will also be collected: dominant hand pain at night; hand stiffness; MAPHAND; hand condition severity scale; how their hands are in comparison to 12 weeks ago, i.e. before receiving gloves (much better/better/no change/worse/much worse). The trial manager will complete the minimal dataset on a 12-week questionnaire (with participant’s PIN included), identifying on the front page that it is a minimal dataset completed by telephone. If the trial manager is unable to obtain this data, this will be recorded in the Trial Database and data entered as missing.

### Data management

#### Data transfer from the University of Salford to CTU

The trial manager will ensure copies of all completed baseline and completed/corrected 12-week questionnaires are provided, in a timely and secure manner (e.g. copies of the questionnaires will be scanned and sent by e-mail using encrypted PDF), to the CTU for data entry. PDF scans of the questionnaires will be securely stored in password protected restricted access folders on the University of Central Lancashire network.

A written Data Management Plan (DMP), containing more detail about the Data Management procedures is available on request from the research team.

Baseline and 12 week questionnaires will be securely stored at the CTU following CTU procedures. At the end of the study, all electronic copies of participant questionnaires will be deleted and purged from the CTU network. Any paper questionnaires and documents (originals or copies) will be securely transferred to the University of Salford. Interview recordings will be deleted following transcription and analysis. Recordings and transcriptions will be stored on a secure server at the University of Salford. All data will be archived for three years in the Centre for Health Sciences Research, University of Salford. Quantitative data will become available to other researchers, on request, following completion and publication of the trial.

### Statistical methods

Primary effectiveness analyses will follow a pre-specified statistical analysis plan and will include the intention to treat (ITT) population. The primary analysis will use multiple linear regression to estimate the effect of group allocation on hand pain during activity, controlling for the stratification variable in the randomisation process (recent DMARD changes) and for the baseline value of the hand pain score. Secondary analysis will repeat the primary analysis method for all other outcomes, using appropriate modelling approaches (i.e. multiple linear regression, logistic regression or ordinal logistic regression, controlling for the stratification variable and the baseline value of the specific measure. Sensitivity analyses will assess for any potential bias in the analysis of the primary outcome measure by excluding participants who were not given gloves, were given the wrong gloves, or who received steroids (oral or injection) between randomisation and outcome measurement (per protocol populations). Data will be analysed by person and using dominant hand results, as reported by the participant. No interim outcome analysis or sub group analysis will be undertaken. We will not undertake any data imputation for the primary analysis. We will undertake multiple imputation of the primary outcome as a sensitivity analysis. Questionnaire responses on medication use, other treatments, perceived benefit of gloves, continued glove use, adherence and occupational therapist treatment record data will be reported as numbers/proportions or means/SD, with 95% confidence intervals. Analyses will be undertaken in Stata version 14 or later [33].

#### Economic evaluation

The economic analysis will include costs to health and social care service providers and health benefits to patients. The time horizon for the evaluation is 12 weeks as per the scheduled trial follow-up. As such neither costs nor outcomes will be discounted. The data will be analysed on an intent-to-treat basis.

The measure of health benefit for the primary economic analysis will be quality adjusted life years (QALYs). This will be estimated from the EQ-5D-3 L and associated utility tariffs. Secondary analysis will explore the cost-effectiveness of arthritis gloves using the primary clinical outcome measure, change in hand pain during moderate activity between baseline and follow-up. The direct costs of healthcare resources used during the trial will be estimated by combining the level of use reported by participants with the unit cost specific to that resource. The unit costs will be derived from published national average unit cost data, the price year will be 2016. Total direct costs will also include the cost of the gloves. Regression analysis will be used to estimate the net total costs and economic benefits of the arthritis gloves compared to the placebo gloves, adjusting for baseline values and the stratification variable (recent DMARD changes).

Cost-effectiveness will be measured as an incremental cost-effectiveness ratio (ICER) for the arthritis gloves versus the placebo gloves. As recommended by NICE for health technology appraisals [34], cost-effectiveness acceptability analysis will be conducted. Non-parametric bootstrapping of the incremental costs and outcomes (estimated from the regression analysis) will be used to estimate the probability that arthritis gloves are cost-effective compared to placebo gloves.

### Data monitoring

The trial will not have a separate Data Monitoring Committee (DMC), as the safety risks associated with this study are very low. Consequently, there are no stopping rules for safety. The project will be overseen by a Trial Steering Committee (TSC), which will perform this function.

### Harms

There is minimal risk or discomfort associated with the intervention (Isotoner) gloves. If a participant has increased hand swelling due to an exacerbation of their condition after glove provision, this can affect fit and potentially cause tingling or numbness. The verbal and written instructions provided during glove provision emphasise that the participant should: regularly check their hands; not wear the gloves continually; stop wearing the gloves if they experience any discomfort or pain or there is any redness or skin reaction; and contact the occupational therapist if they experience any problems.

Glove use will be modified or discontinued by the treating occupational therapist only if an adverse event related to glove wear causes concern. This may occur at the review appointment or if the participant contacts the occupational therapist with concerns. Adverse events caused by arthritis gloves have been identified in clinical practice and therapists modify the wear regimen as applicable. Adverse events and modifications include:

- Numbness; pins and needles; and/or fingertips becoming discoloured (i.e. they go red, white or blue) during glove wear. This may be due to gloves being too tight. A larger glove may be fitted if this occurs.
- Allergic reaction or skin irritation can occasionally occur. Gloves are either left off for a time and re-tried or discontinued.
- Sleep disturbance at night. This can occur because the gloves feel hot or itchy. Participants are recommended to wear the gloves for part of the night or leave them off when necessary.

It is unlikely any harm will arise from participants wearing placebo gloves as most adverse events occur due to pressure effects.

Any adverse event considered by the occupational therapist to be resulting from glove wear, or any other minor problems related to glove wear, will be recorded by the occupational therapist on the A-Gloves Occupational Therapy Treatment Record Form at the review appointment and a copy returned to the CTU Data Manager.

### Auditing

The trial manager will conduct at least one on-site monitoring visit per year over the course of the study to all clinical sites to educate, support and solve problems. The trial manager will provide the research facilitator/principal investigator with a site file checklist prior to the visit and ask them to ensure the site file is up-to-date. The trial manager will check the completed checklist and site file at the visit. There will also be central monitoring, by the CTU and trial manager, of quality of source documentation, including consent form completion and adverse event reporting.

The Chief Investigator will permit study-related monitoring, audits and inspections by the Ethics Committee, lead R&D department, the University and any NHS Trust Research Governance Managers requiring this. The study will be monitored in accordance with NHS and University Research Governance procedures. The Chief Investigator will ensure that any regulatory authority is given access to all study related documents and study related facilities. Principal investigators will be asked to audit their site files at the beginning and end of the study using a checklist provided by the University Research Centre and to allow study related monitoring, audits and inspections as above.

### Composition roles and responsibilities of: the co-ordinating centres, TMG and TSC and data analysis team

Management of the trial is joint between the Centre for Health Sciences Research, University of Salford (Hammond, Prior) and Lancashire Clinical Trials Unit (Sutton). Prof Hammond (Chief Investigator), Dr Prior (Trial Manager) at the University of Salford, in conjunction with advice and support from Dr Sutton (Lead, Lancashire CTU) and other CTU staff, as appropriate, and Dr Cotterell (Statistician) will be responsible for: (i) writing the study protocol and any revisions, obtaining all study approvals and any amendments, preparation of study documentation, study planning, contributing to Trial Management Group and Trial Steering Committee meetings, producing interim and final reports to the funder and approving agencies (ethics committees, Trust R&D departments) (ii) Serious Adverse Event reporting (iii) Trial master file management and ensuring sites have site files, and content is monitored periodically (iv) Budget administration in conjunction with Salford Royal NHSFT (Research & Development) and University of Salford (Contracts Officer, Research and Innovation) (v) Advice for Principal Investigators, Research Facilitators and occupational therapists s (vi) Site initiation visits with Research Facilitators/occupational therapists/Principal Investigators (vii) Organising occupational therapy training in glove provision and study procedures and day-to-day management of the project. The Trial Management Group (TMG) consists of AH (Chair) and the protocol contributors (listed in author contributions), and YP as the trial manager). The TMG will approve documentation, study protocol procedures, advise on ethics application, monitor trial progress by reviewing the trial progress reports, any problems arising, be advised of any Serious Adverse Events, review findings and plan dissemination. The TMG will meet at 6 monthly intervals and receive reports of trial progress. Teleconferences and ad-hoc meetings will be held during the study if issues arise requiring discussion. The Trial Steering Committee (TSC) consists of: Dr Peter Klimiuk, Consultant Rheumatologist, Pennine Musculoskeletal Partnership Ltd, Oldham (Chair); Cathy Ball, Research Clinical Specialist Hand Therapist, Kennedy Institute for Rheumatology, Oxford; Dr Michael Callaghan, Research Fellow, University of Manchester and Clinical Specialist Physiotherapist, Manchester Royal Infirmary; Mike Bradburn, Senior Medical Statistician, Sheffield Clinical Trials Unit, SCHARR, University of Sheffield. The TSC will meet three times and also act as the Data Monitoring Committee (DMC) and provide trial oversight and data monitoring. The TSC will meet to approve the protocol, advise on procedures and progress, data monitoring and review findings and monitor trial progress by reviewing trial progress reports. The project does not have a DMC, as the safety risks associated with this study are very low. The TSC will have access to the unblinded data on completion of the analysis. No interim analyses are planned.

### Protocol amendments

Any subsequent modifications to the protocol which may impact on the conduct of the study, potential benefit to the patient or may affect patient safety (including changes of study objectives, study design, patient population, sample sizes, study procedures, or significant administrative aspects) will require a formal amendment to the protocol. Such amendment will be agreed by TMG and TSC members and approved by the approving NRES Ethics Committee prior to implementation and notified to the participating Trust R&D departments in accordance with local regulations.

Administrative changes of the protocol are minor corrections and/or clarifications that have no effect on the way the study is to be conducted. These administrative changes will be agreed upon by TMG members and documented in a Memorandum filed in the trial master file. The approving NRES Ethics Committee may be notified of administrative changes at the discretion of the TMG. Protocols and documents will be version controlled.

### Dissemination

Findings will be submitted to rheumatology and health professional conferences and journals. A summary of findings will be provided to relevant health professional and arthritis patient organizations, requesting these are included in websites and newsletters. We will also produce guidelines, for both therapists and patients, on how to correctly fit and wear arthritis gloves, based on the A-GLOVES Trial Occupational Therapy Glove Provision Manual [17] and disseminate these as above.

## Discussion

This protocol describes a definitive, pragmatic, patient-blinded, and multi-centre superiority randomised parallel group trial, which aims to determine the effectiveness and cost-effectiveness of the use of arthritis gloves for people with RA or IA with persistent hand pain. The results of this trial will inform the evidence base to support the prescription of arthritis gloves for people with RA or IA and hand pain. The results of this study will be published as soon as they become available.

## Additional file

Additional file 1: A-GLOVES: Testing Arthritis Gloves in Rheumatoid/Inflammatory Arthritis. (DOC 446 kb)
