# Supplementary material for: The effects of arthritis gloves on people with Rheumatoid Arthritis or Inflammatory Arthritis with hand pain: a study protocol for a multi-centre randomised controlled trial (the A-GLOVES trial)
Source: BMC Musculoskelet Disord. 2017 May 30;18:224. doi: 10.1186/s12891-017-1583-4 (PMC5450242; doi:10.1186/s12891-017-1583-4)
Supplement: Additional file 1: — A-GLOVES: Testing Arthritis Gloves in Rheumatoid/Inflammatory Arthritis. (DOC 446 kb) [file 12891_2017_1583_MOESM1_ESM.doc]

| 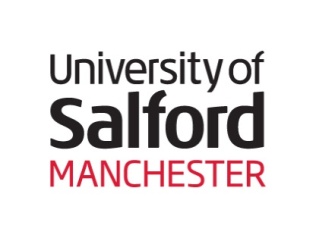 | 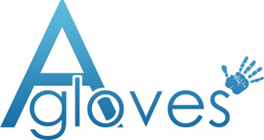 | [hospital/site headed paper] |
| --- | --- | --- |

**PARTICIPANT INFORMATION SHEET:**

**A-GLOVES: Testing Arthritis Gloves in Rheumatoid/Inflammatory Arthritis**

**Part One: Introduction**

We would like to invite you to take part in a research study. The study is being run by the Centre for Health Sciences Research, University of Salford. The Lancashire Clinical Trials Unit (CTU), (University of Central Lancashire) is also supporting the trial. Before you decide, you need to understand why the research is being done and what it will involve for you. Please take time to read the following information carefully. Talk to others about the study if you wish. The research facilitator at your hospital will be happy to go through this information sheet with you. They can answer any questions you have. Alternately, our **Trial Manager, Dr Yeliz Prior** (0161 2950211 / 07471 826719 and [y.prior@salford.ac.uk](mailto:y.prior@salford.ac.uk)) can also do this on the telephone. Please ask if there is anything that is not clear to you or if you would like more information. Take time to decide whether or not you wish to take part.

**What is the purpose of this study?**

People with arthritis can have problems with hand pain, swelling and/ or stiffness. Occupational therapists may recommend they try wearing arthritis gloves. These might help relieve hand symptoms. Your Consultant Rheumatologist, Rheumatology nurse or occupational therapist helped identify this study might be relevant for you. We are testing two types of arthritis gloves to see if they are helpful or not for people with Rheumatoid or Inflammatory Arthritis.

We would like you to try wearing **one** pair of these gloves for up to 12 weeks. Which type of gloves you are given is randomly allocated by a computer. An occupational therapist fits these gloves for you.

You are asked to complete two questionnaires. One is completed at the beginning of the study, before you are given the gloves. The second is completed 12 weeks later, to find out if these arthritis gloves helped you or not.

The two types of arthritis glove we are testing look like this:

| 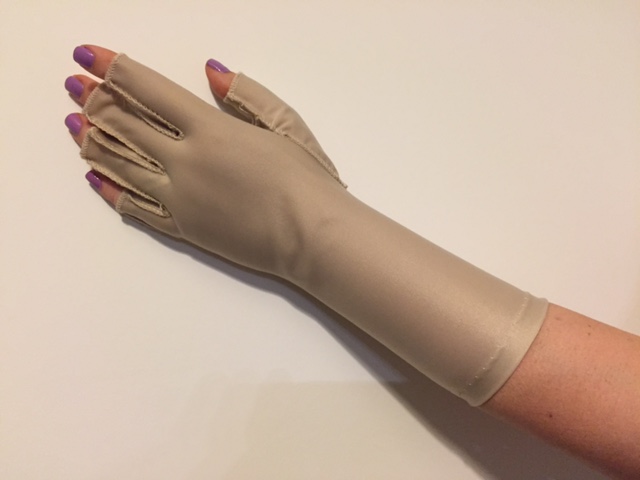 | 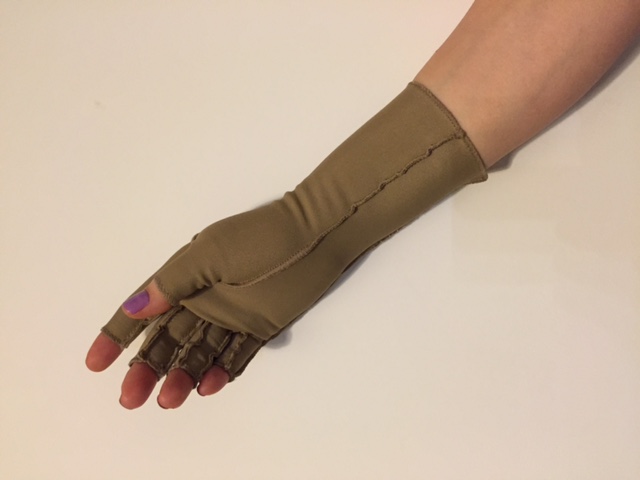 |
| --- | --- |

Both types of gloves leave the finger ends free. You can still use your hands as normal. The therapist discusses with you when it is best for you to wear these (during the day and/or night; how long for; for what activities). They are **not** worn all the time. You can leave them off at times when you need to. The gloves are washable.

There is no good quality research about whether these arthritis gloves work. Several small studies, done over 25 years ago, tested gloves worn at night by people with rheumatoid arthritis. Some studies found they helped and others did not. Since then, gloves have changed, treatment of arthritis has changed, gloves can be worn day and/or night; and a wider variety of people with arthritis are offered gloves. People with arthritis may find them helpful. We want to see whether these gloves have any effect. We are asking for your help with this study.

**Why have I been invited?**

We are asking people to take part who have:

1. **One** of two types of arthritis:

- Inflammatory arthritis (IA) or
- Rheumatoid arthritis (RA).

2. **Persistent** pain in your hand/s causing you one or more of the following:

- difficulty using your hand/s in the day
- disturbed sleep
- difficulty using your hand/s first thing in the morning

3. **Not** previously worn arthritis gloves and are willing to try wearing arthritis gloves in this study.

A member of your health care team identified this study may be relevant for you

**Do I have to take part?**

It is up to you to decide. Take your time to do so. Please discuss it further and ask questions with the person giving you this information. If you want to ask anything further, please call our **Trial Manager, Dr Yeliz Prior, on 0161 295 0211 or 07471 826719** (e-mail: y.prior@salford.ac.uk). Dr. Prior will be happy to answer any questions. (You can also send her the Contact Details Form in the FREEPOST envelope enclosed, if you are interested and want more information. Dr Prior can then call you).

If you decide not to take part, this will not affect the standard of care you receive. If you decide to take part, you can still withdraw at any time. You do not need to give a reason. This will not affect the standard of care you receive.

**What will happen to me if I take part?**

1. **If you decide to take part:** we will ask you to sign a consent form to show you agreed. The research nurse or occupational therapist at your hospital will complete the consent form with you.

If you cannot come to the hospital to complete the consent form, you can complete consent by telephone and mail with the A-GLOVES Trial Manager, Dr Yeliz Prior. Dr Prior would need your contact details to do this. The research nurse or occupational therapist, discussing the study with you, will **only** send your contact details to Dr Prior **if** you agree to this. Or please mail your contact details yourself to Dr Yeliz Prior on the Contact Details Form enclosed, using the FREEPOST envelope. Dr Prior will call you. She can then complete consent by telephone and mail with you.

2. Following consent, we will give/send you a questionnaire to complete at home. This questionnaire will ask about, for example, your age, gender, diagnosis and medication. It will also ask about your hand pain and stiffness, hand function and other health-related information. The questions are mostly answered using tick boxes/ circling numbers. It takes about 30 minutes to fill in. You do this in your own time at home. Then you post it back to us in the FREEPOST envelope provided (i.e. at no cost to yourself).

3. After you return the questionnaire, within 3 weeks you should receive an appointment with an occupational therapist. This will be at the Rheumatology /occupational therapy department you normally attend. The therapist will provide you with the arthritis gloves. We have trained the therapist to fit the gloves following our study instructions. You are asked to wear the same gloves you are fitted with for up to 12 weeks, as recommended. The therapist will discuss and agree with you when it is best for you to wear them. Sometimes, some people find gloves are itchy or hot if worn at night, or may have some tingling in the fingers. These feelings stop when you take the gloves off. The therapist will explain what to do should you have any minor problems. We ask you not to wear any other type of arthritis gloves during the 12 weeks of the study. After you have had the gloves for 2 to 4 weeks, the therapist will check the gloves with you. This will be at the hospital or may be by telephone. If you have any problems with or concerns about wearing the gloves before then or after, just contact the therapist.

4. At the end of the 12 weeks, we will send you a second questionnaire. This again asks about: your hand pain, stiffness, hand function and other health-related information. It also asks about: any changes in your medication and any other treatment you had in the last 12 weeks (such as physiotherapy). It asks how your hands are compared to before getting the gloves; whether you think the gloves help or not, and if you had any problems with them. This takes about 30 minutes. You do this in your own time at home. Then you post it back to us in the FREEPOST envelope provided.

5. Finally, we also want to interview **20-30 people**. You can let us know if you would like to be interviewed on the consent form. If you prefer not, it’s not a problem. We don’t need everyone to help. If you do agree to the interview, you can still change your mind at any time. In the interview, we ask your views about the gloves. For example: any benefits or negative effects of glove wear; and glove appearance and quality. The interview will be at a time and place that suits you. This can be in your own home or the hospital at which you were given your arthritis gloves. We would like to audio-record it to help us later in writing down accurately what you said. The recordings are only listened to by our research staff. Your name is not identified when these are typed up. These are only read by the researchers. We delete the recordings once the content is written down. We will not ask about any private information. Please do not disclose such information to the interviewer.

After you have completed the second questionnaire (and interview if you choose to do this), then we will send you a letter. This explains what to do about using the arthritis gloves in future. If you do not wish to keep them, simply throw them away in your household waste. They are not re-usable. If you want to try a different type of arthritis glove after you finish the study, please contact the OT.

**Expenses and payments**

There is no expense in completing the questionnaires, as we provide FREEPOST envelopes for the reply. We will pay back any travel costs you have attending clinics or occupational therapy to complete consent, to get the gloves and/ or for an interview. We provide a claim form. Please attach any receipts (for bus, train, parking) to this. Include the number of miles travelled if you come by car.

**What are the possible benefits of taking part?**

The study itself will not directly help you now, although you may find the gloves provided help you. The information you give us will help us understand any effects arthritis gloves may have on hand pain and function, and whether one type of arthritis glove is better than the other. In future, this will help therapists plan treatment for people with arthritis.

**What are the possible disadvantages and risks of taking part?**

The gloves are provided and fitted for your use by an occupational therapist at the hospital. The therapist has received training in fitting the gloves. We do not expect there are any disadvantages or risks to you in taking part in the study. If you choose to take part in the interviews, we will arrange these at a place and time to suit you.

*If the information in Part 1 has interested you and you are considering taking part, please read the additional information in Part 2 before making any decision.*

**Part Two: Further information**

**Will my taking part in this study be kept confidential?**

Yes. We will follow ethical and legal practice. We will handle all information about you in confidence. We will store it securely at the University of Salford and the Lancashire Clinical Trials Unit. All information which is collected about you during the course of the research will be kept strictly confidential. We only identify you in the interview recording by a number. You have the right to check the accuracy of the data held and correct any errors. Only staff involved in the research study see your information. Any direct quotes from the audiorecordings we use in reports, will not identify you in any way. We send a copy of your consent form to the hospital Consultant responsible for your care. This will be placed in your medical records.

**What will happen if I don’t want to carry on with the study?**

You can withdraw from the study at any time, and your care will not be affected. However, we will use the data collected up until your withdrawal unless you tell us not to.

**What will happen if there is a problem?**

If you have problems with the arthritis gloves, please contact and discuss this with the occupational therapist who gave them to you. If you have queries about the study, questionnaires or interview, please contact our Trial Manager (Dr Yeliz Prior) who will answer these.

If you have any complaint about the way you have been dealt with during the study, or any possible harm you might suffer, we will address. If you have a concern about any aspect of this study, you should ask to speak first to: the occupational therapist who provided the arthritis gloves to you and the researchers. We will do our best to answer your questions (contact our Trial Manager: Dr Yeliz Prior on Tel: 0161 295 00211 or e-mail: y.prior@salford.ac.uk). If you remain unhappy and wish to complain formally, you can do this through the NHS Complaints Procedure. Details can be obtained from:

**The Customer Care Manager**

Address of the participating hospital

Tel: xxxx

**What will happen to the results of the research study?**

We will send you a summary of the findings when the study is fully completed. When you finish taking part, we will send you a letter saying when this is likely to be.

We will write up the findings and submit them for publication. This will help Rheumatology and Occupational Therapy departments learn from our experiences. You will not be identified in any report or publication. We will send your occupational therapist a summary of the study when it is finished. We will also aim to present the study results at national and international rheumatology conferences. We will inform arthritis charities so they can choose whether to make the findings available to other people with arthritis via their websites/ magazines.

**Who is organizing and funding the research?**

The Centre for Health Sciences Research, University of Salford and the Lancashire Clinical Trials Unit are organizing this trial. We are working with the Occupational Therapy and Rheumatology departments at: Stepping Hill Hospital (Stockport), St Helens Hospital, Pennine Musculoskeletal Partnership Limited (Oldham), Trafford Hospital (Manchester), Leighton Hospital (Crewe), Victoria Infirmary (Northwich), Southport and Formby District General Hospital, Hexham General Hospital, North Devon District Hospital, Royal Hallamshire Hospital (Sheffield), Cannock Chase Hospital, New Victoria Hospital (Glasgow), Singleton Hospital (Swansea), Scunthorpe General Hospital, King’s Mill Hospital (Sutton-in-Ashfield) and North Manchester General Hospital. We are also working with: Haywood Hospital (Stoke-on-Trent), Chapel Allerton Hospital (Leeds) and St Albans City Hospital/ Hemel Hempstead Hospital.

The study is funded by the **National Institute for Health Research (NIHR) through a Research for Patient Benefit (RfPB) grant.** The Chief Investigator is Professor Alison Hammond.

**Who has reviewed the study?**

All research in the NHS is looked at by independent groups of people, called a Research Ethics Committee, to protect your safety, rights, wellbeing and dignity. This study has been reviewed and given favorable opinion by North of Scotland Research Ethics Committee 2. The Research Ethics Committee at the University of Salford and University of Central Lancashire have also approved the study.

**Further information and contact details**

If you have any questions about the study, wish to discuss taking part or have any concerns; please contact the researchers leading the study:

**Dr Yeliz Prior** **– A-GLOVES Trial Manager**

Centre for Health Sciences Research, University of Salford

L701 Allerton, Frederick Road, Salford M6 6PU

0161 295 0211 – 07471 826719

[y.prior@salford.ac.uk](mailto:y.prior@salford.ac.uk)

**Professor Alison Hammond – Chief Investigator**

Centre for Health Sciences Research, University of Salford

L701 Allerton, Frederick Road, Salford M6 6PU

Tel: 0161 295 0038 – [a.hammond@salford.ac.uk](mailto:a.hammond@salford.ac.uk)

Information about the study is also available from our website: [http://www.salford.ac.uk/rehabilitation-research/a-gloves-study](https://uos-portal.salford.ac.uk/owa/redir.aspx?C=RX8LuN2kD0asamqQvL777Qx5wD9UiNIIKJu0gC4wE7qFgBhxq9GHa5MIOZnI3E3HQdRLQpec1-g.&URL=http%3A%2F%2Fwww.salford.ac.uk%2Frehabilitation-research%2Fa-gloves-study)

If you have any general queries about participating in research you can contact your hospital’s Patient Advisory and Liaison Service (PALS). General information is also available on the Involve website ([www.invo.org.uk](http://www.invo.org.uk/)). INVOLVE is a national advisory Group, funded by the Department of Health, which supports active public involvement in NHS, public health and social care research.

In the event that something does go wrong and you are harmed during the research and this is due to someone’s negligence then you may have grounds for a legal action for compensation against the NHS Trust at which you received the gloves and/or the University of Salford but you may have to pay your legal costs. The normal National Health Service complaints mechanisms will still be available to you (if appropriate).

**Finally**

Thank you for considering taking part. Please keep this information sheet. We will ask you to sign a consent form if you agree to take part. We will give you a copy of this to keep.

| 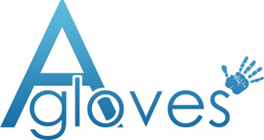 | 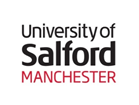 | [Hospital/site heading] |
| --- | --- | --- |

**Site Number Participant ID:**

|  |  |  |  |  |  |
| --- | --- | --- | --- | --- | --- |

**A-GLOVES** **CONSENT FORM**

**Title of project: A-GLOVES: Testing Arthritis Gloves in Rheumatoid/Inflammatory Arthritis**

**Name of researcher: Prof Alison Hammond Please INITIAL all boxes (i.e. do not tick)**

1. I confirm that I have read and understand the information sheet dated 8.7.15 (Version 1) for the above study. I have had the opportunity to consider the information, ask questions and have had these answered satisfactorily.
2. I understand that my participation is voluntary and that I am free to withdraw at any time, without giving any reason, without my medical care or legal rights being affected.
3. If I do later choose to withdraw from the study, I agree that any data collected up to that point can be kept and used in the study.
4. **Optional:** I agree to take part in the face-to-face interview about the gloves. I understand that the interview will be audio-recorded, recordings will be deleted once transcribed and anonymised quotes may be given verbatim in reports.
5. I understand that relevant sections of my medical notes may be looked at by regulatory authorities or from the NHS Trust, where it is relevant for my taking part in this research. I give permission for these individuals to access my records.
6. I agree to my Rheumatology Consultant, being informed of my participation in this study.
7. I understand a copy of this form and my contact details will be forwarded to the research team at the University of Salford.
8. I agree to take part in the above study.

Name of patient:_____________________Date:____________**Signature:**_________________

Name of person:

taking consent:______________________ Date:____________ **Signature**:________________
